# Unravelling lung adenocarcinoma in an immunocompetent patient with endobronchial aspergilloma: A case report

**DOI:** 10.1002/rcr2.1409

**Published:** 2024-06-10

**Authors:** Khai Lip Ng, Nai‐Chien Huan, Wei Loon Tan, Nur Husna Mohd Aminudin, Fazilah Hassan, Kasuma Mohamed Nordin

**Affiliations:** ^1^ Division of Respiratory Medicine, Department of Medicine Melaka Hospital Melaka Malaysia; ^2^ Department of Respiratory Medicine Queen Elizabeth Hospital Kota Kinabalu Malaysia; ^3^ Department of Pathology Melaka Hospital Melaka Malaysia

**Keywords:** endobronchial aspergilloma, immunocompetent, lung adenocarcinoma

## Abstract

Inhalation of Aspergillus spp. can cause a wide spectrum of lung diseases. Endobronchial aspergilloma is an uncommon clinical entity that occurs because of Aspergillus spp. overgrowth in the airway lumen. We present a 73‐year‐old gentleman with a rare dual pathology of endobronchial aspergilloma and endobronchial adenocarcinoma. He initially presented with prolonged cough, dyspnoea, and haemoptysis. Bronchoscopy revealed an obstructed right main bronchus by a necrotic mass whereby histological examination showed evidence of Aspergillus spp. infection. The lesion however persisted despite treatment with anti‐fungal agents. Repeated bronchoscopy and biopsy eventually unravelled an underlying endobronchial adenocarcinoma. He received chemotherapy but ultimately passed away 3 months later.

## Graphical abstract

We present a 73‐year‐old gentleman with a rare dual pathology of endobronchial aspergilloma and endobronchial adenocarcinoma who initially presented with prolonged cough, dyspnoea, and haemoptysis. Bronchoscopy revealed an obstructed bronchus by a necrotic mass whereby histological examination showed evidence of *Aspergillus* spp. infection which persisted despite treatment with anti‐fungal agents. Repeated bronchoscopy and biopsy eventually unravelled an underlying endobronchial adenocarcinoma.

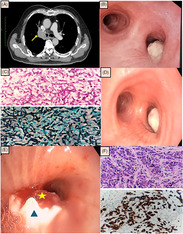

## INTRODUCTION

*Aspergillus* spp. is a ubiquitous fungus that can be found in soil, water, and decomposing matters.^1^ Inhalation of Aspergillus spores can cause a broad spectrum of lung diseases, depending on patients' immune status and underlying structural lung diseases.^1^, ^2^ Endobronchial aspergilloma is an uncommon clinical entity that occurs due to Aspergillus spp. overgrowth in the airway lumen. It is not well described in the literature and is usually not classified along with other forms of pulmonary aspergillosis.^1^, ^3^ Coexisting endobronchial aspergillosis and endobronchial adenocarcinoma is rare. Herein, we describe an immunocompetent elderly gentleman with right main bronchus obstruction by endobronchial aspergilloma. The lesion persisted despite treatment with anti‐fungal. Repeated bronchoscopy and biopsy eventually revealed an underlying lung adenocarcinoma.

## CASE REPORT

**FIGURE 1:**
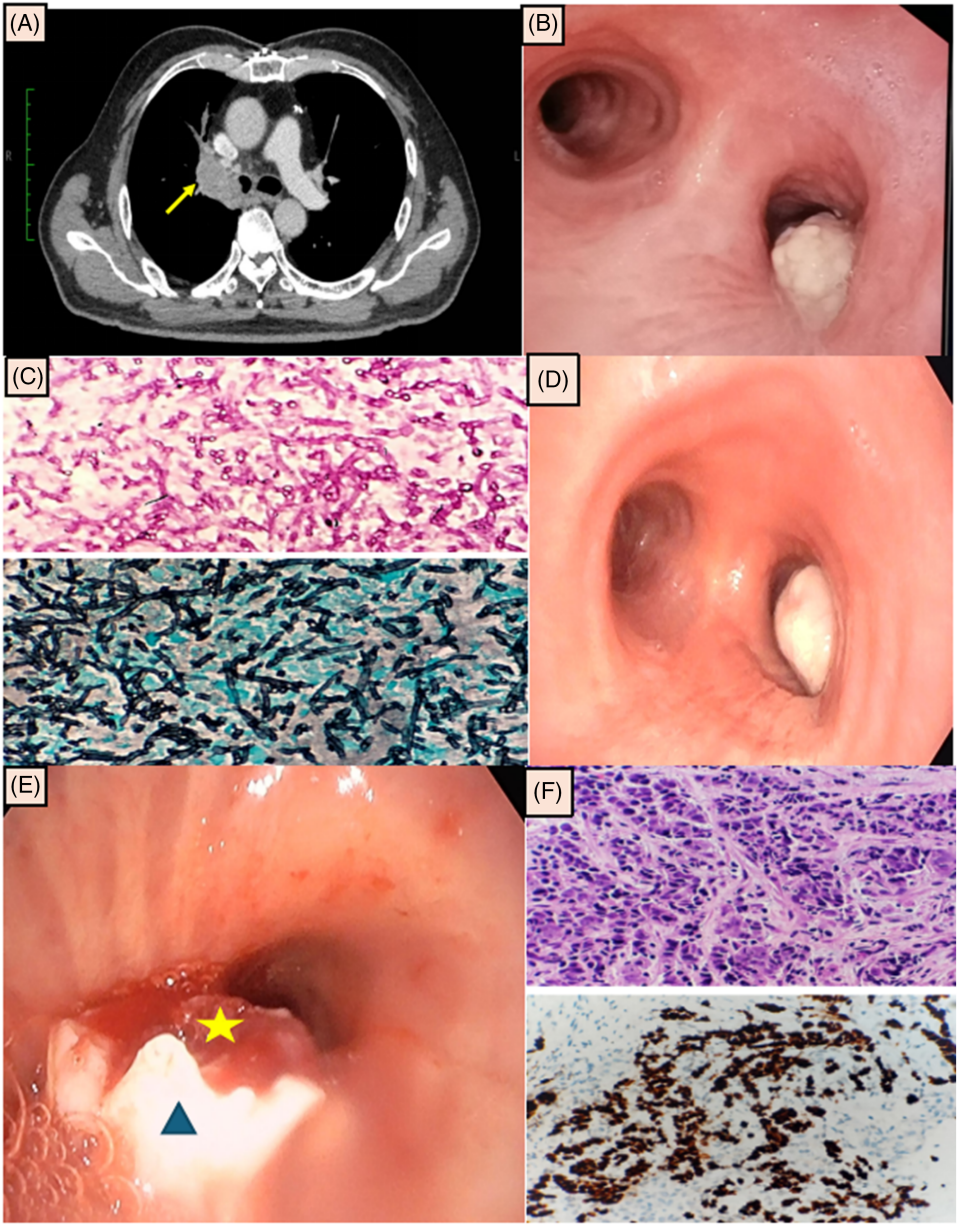
(A) Computed tomography (CT) of the thorax showed a right hilar soft tissue lesion (yellow arrow) with extension into the right main bronchus. (B) Flexible bronchoscopy showed a whitish necrotic mass which obscured about 60% of the right main bronchus lumen. (C) Histopathological examination showed fungal hyphae (acute angle, or dichotomous branching, septate hyphae) on Periodic Acid‐Schiff (PAS) and Grocott Methenamine Silver (GMS) stain. (D) Despite systemic antifungal therapy, the endobronchial lesion at the right main bronchus progressed on repeated bronchoscopy. (E) An endobronchial mass (yellow star) was seen after removing the outer necrotic layers (blue triangle). (F) Histopathological examination of the inner layer of the mass showed enlarged, moderately pleomorphic nuclei, with prominent nucleoli. Immunohistochemistry stains were positive for TTF‐1. The overall picture is consistent with lung adenocarcinoma.

A 73‐year‐old gentleman with diabetes mellitus, ischaemic heart disease and chronic obstructive pulmonary disease (COPD, GOLD E) presented with a one‐month history of productive cough, dyspnoea, and haemoptysis. He has been an active cigarette smoker for 60 pack years. Chest radiograph showed a thickened right paratracheal stripe with right hilar opacity. Computed tomography (CT) of the thorax demonstrated multiple right upper lobe lung nodules and a right hilar mass which extended to the right main bronchus with bilateral mediastinal lymphadenopathies (Figure 1A). A necrotic mass that occluded approximately 60% of the right main bronchus was seen during flexible bronchoscopy (Figure 1B). Multiple superficial and deep forceps biopsies led to the recanalisation of the right main bronchus. We did not use a cryoprobe during the procedure as forceps biopsy itself was successful in restoring patency of the airway. Besides, we were concerned about a potentially higher bleeding risk with cryobiopsy as the procedure was done under sedation without artificial airway. Histopathology was negative for malignancy but many dichotomous branching and septate hyphae were seen, suggesting Aspergillus spp. (Figure 1C). Fungal culture from the biopsy grew *Aspergillus fumigatus*. Cultures for bacteria and tuberculosis were negative. He was commenced on a two‐week course of intravenous amphotericin‐B followed by 6 weeks of oral voriconazole.

Nevertheless, he had ongoing dyspnoea and lost further weight despite being on antifungal agents. Another CT thorax 6 weeks after therapy revealed a persistent right hilar mass. Repeated bronchoscopy showed a recurrence of necrotic endobronchial mass which caused almost total occlusion of the right main bronchus (Figure 1D). An endobronchial mass was seen after the outer necrotic layers were removed with forceps (Figure 1E). Histopathological examination of the outer necrotic layer displayed fungal bodies, while the inner layer endobronchial mass showed lung adenocarcinoma (Figure 1F). No driver mutation was detected. He received chemotherapy, which was complicated by neutropenic sepsis. He opted for conservative care and passed away at home after 3 months.

## DISCUSSION

Endobronchial aspergillomas have been reported in patients with benign and malignant endobronchial tumours such as lipoma,^4^ carcinoid,^5^ and lung cancer.^6^, ^7^ Earlier reports^7^ hypothesized that *Aspergillus* spp. can predispose to cancer development although further confirmative studies are needed. In our patient, we postulate that endobronchial invasion by lung cancer caused airflow stasis and disrupted the clearance of airway secretions, which promoted *Aspergillus* spp. colonization. Deposits of cellular debris and necrotic tissues due to rapid lung cancer cell growth provided a favourable thriving environment for *Aspergillus* spp. Endotoxins and proteolytic enzymes produced by *Aspergillus* spp. further aggravated airway tissue necrosis.^5^, ^8^, ^9^ No consensus has yet emerged as to whether endobronchial aspergilloma should be classified as an invasive form of Aspergillus lung disease. Ma et al. described it as a non‐invasive form akin to the endobronchial form of ‘fungal ball’ without clear lung parenchyma invasion.^2^ Wu et al., on the other hand, viewed Aspergillus tracheobronchial disease as a form of invasive aspergillosis. Four subtypes were described: superficial infiltration (type 1), full layer involvement (type 2), occlusion type (type 3), and mixed type (type 4).^8^ Our patient's bronchoscopy appearance is consistent with the type 3 classification. It remains unclear whether the subtypes of endobronchial aspergilloma can be affected by factors such as Aspergillus spp. fungal load, host immune status, and/or airway characteristics.

Diagnostic delays are common in patients with lung cancers coexisting with *Aspergillus* spp., as the tumour cells can be covered by thick outer layers of endobronchial aspergilloma. An earlier review by Nillson et al. showed that 16 out of 31 patients with primary lung cancer coexisting with *Aspergillus* spp. received prolonged courses of anti‐fungal treatment before anti‐cancer therapies were eventually given.^5^ Furthermore, well‐known risk factors for invasive Aspergillus spp. infection such as neutropenia, bone marrow transplantation, cytotoxic therapy and haematological malignancies are not always observed in patients with endobronchial aspergilloma.^10^, ^11^ Our patient underwent two separate bronchoscopy procedures interspaced with a prolonged course of anti‐fungal therapy before a final diagnosis of lung malignancy was made.

Optimal treatment strategies for endobronchial aspergilloma have not been established. Polarized strategies have been proposed: some authors promote early bronchoscopy intervention coupled with systemic antifungal therapies^8^, ^12^ while others advocate observation without treatment among stable patients, given the presumed non‐invasive nature of endobronchial aspergilloma.^2^, ^13^ Among patients with coexisting lung cancer and aspergillosis, prolonged antifungal treatment can delay anticancer therapy, allowing for cancer disease progression. Besides, anti‐cancer therapies such as chemotherapy and radiotherapy can compromise the immune system and can trigger the transition of *Aspergillus* spp. from commensalism to a pathogenic phase such as invasive aspergillosis.^9^ Gupta et al. described a patient who developed invasive aspergillosis after receiving immunotherapy treatment for lung cancer.^14^

In conclusion, endobronchial aspergilloma is a rare clinical condition that can occur in the setting of lung cancer. Early detection followed by effective endobronchial biopsies can confirm the diagnosis. Treatment strategies for endobronchial aspergilloma with coexisting lung cancer are not well defined. Bronchoscopy interventions coupled with systemic antifungals followed by anti‐cancer therapies are logical management steps.

## AUTHOR CONTRIBUTIONS

Khai Lip Ng, Nai‐Chien Huan, Wei Loon Tan contributed to the design and implementation of the case report. Khai Lip Ng, Nai‐Chien Huan, Wei Loon Tan wrote the manuscript. Khai Lip Ng and Nur Husna Mohd Aminudin carried out the procedures and treatment mentioned. Fazilah Hassan interpreted the pathological slides. Kasuma Mohamed Nordin supervised the project. All authors discussed the study and contributed to the final manuscript.

## CONFLICT OF INTEREST STATEMENT

None declared.

## ETHICS STATEMENT

The authors declare that appropriate written informed consent was obtained for the publication of this manuscript and accompanying images.

## Data Availability

The data that support the findings of this study are available from the corresponding author upon reasonable request.
